# A Comprehensive Pan-Cancer Analysis of 33 Human Cancers Reveals the Immunotherapeutic Value of Aryl Hydrocarbon Receptor

**DOI:** 10.3389/fimmu.2021.564948

**Published:** 2021-07-05

**Authors:** Zhuomao Mo, Pan Li, Zhirui Cao, Shijun Zhang

**Affiliations:** Department of Traditional Chinese Medicine, The First Affiliated Hospital, Sun Yat-sen University, Guangzhou, China

**Keywords:** aryl hydrocarbon receptor, pan cancer, immunotherapy, immune response, prognosis

## Abstract

**Background:**

Previous studies have reported the potential of aryl hydrocarbon receptor (AhR) in cancer immunotherapy. However, the mechanisms underpinning its therapeutic value have yet to be comprehensively investigated. Thus, this research aimed to explore the underlying association between AhR and cancer immunotherapy in 33 human cancers.

**Methods:**

The gene expression data and clinical characteristics of 33 cancers were retrieved from The Cancer Genome Atlas database. The immunotherapeutic cohorts included GSE67501 and GSE78220 as well as IMvigor210, which were obtained from the Gene Expression Omnibus database and included in a previously published study respectively. Clinical parameters, including patient age, gender, survival, and tumor stage were analyzed to assess the prognostic value of AhR. The activity of AhR was generated by single sample gene set enrichment analysis and used to evaluate the difference between the AhR transcriptome and protein expression level. To better understand the role of AhR in cancer immunotherapy, the correlation between AhR and tumor microenvironment, as well as its relation to immune processes/elements, such as immune cell infiltration, immune inhibitors and stimulators, and the major histocompatibility complex were analyzed. The relevant underlying pathways associated with AhR signaling in cancer were also explored. Furthermore, the correlation between AhR and two immunotherapeutic biomarkers (tumor mutational burden and microsatellite instability) was investigated. Finally, the relationship between AhR and immunotherapeutic response was explored using three independent immunotherapeutic cohorts.

**Results:**

Although AhR was not closely associated with age (5/33), gender (3/33), or tumor stage (3/21) in any of the studied human cancers, it exhibited potential prognostic value for predicting patient survival. Consistency has been observed between AhR activity and expression in some cancers (7/33). Generally, AhR presented a robust correlation with immune cell infiltration, immune modulators, and immunotherapeutic markers. Moreover, high AhR expression was significantly related to immune-relevant pathways. However, no significant correlation was observed between AhR and the immunotherapeutic response.

**Conclusions:**

This research investigated the immunotherapeutic value of AhR in 33 human cancers, providing evidence regarding the function of AhR and its role in clinical treatment. However, considering that a bioinformatics approach was adopted, the current results are preliminary and require further validation.

## Introduction

The aryl hydrocarbon receptor (AhR) is a cytoplasmic receptor and transcription factor that is mainly activated by its cognate ligand (1). Initially, this receptor garnered attention because of its crucial role in mitigating the toxic effects of environmental pollutants (1). AhR belongs to the basic helix-loop-helix/PER-ARNT-SIM transcription factor family and was first studied as a receptor for the exogenous ligand 2, 3, 7, 8-tetrachlorodibenzo-p-dioxin (2). Moreover, it is distributed in almost all human tissues and is highly expressed in the placenta, liver, and the lungs (3).

The physiological effects of AhR activation play a key role in carcinogenesis and immune modulation, as AhR is highly expressed and chronically activated in both hematological malignancies (4, 5) and solid tumors (6–8). Constitutively high AhR expression and nuclear localization is observed in invasive tumor tissues and malignant tumor cell lines (9, 10), which may suggest its involvement in the inflammatory response and cell cycle progression (11). In terms of immune modulation, AhR participates in the modulation of both innate and adaptive immunity (12). Natural killer (NK) cells are an important component of the innate immune system and contribute to the antitumor immune response. By binding to the ligand FICZ, AhR is activated and can thus induce NK cells to secrete interferon-γ and simultaneously enhances their antitumor activity (13). Researchers also proposed that AhR activation in the tumor microenvironment increases the proportion of regulatory T cells based on the immunosuppressive effects of the AhR ligand (1). Generally, AhR has an important role in both tumors and the immune system. However, few studies have actually focused on the immunotherapeutic value of AhR in human cancers.

In this study, the AhR expression landscape of 33 different cancers was presented and the underlying effects of AhR on the tumor immune microenvironment were investigated. Many important immune modulators and dynamic immunological biomarkers, such as tumor mutational burden (TMB) and microsatellite instability (MSI), were investigated in this research. Furthermore, the correlation between AhR expression and immune checkpoint blockade treatment was explored. Overall, this work provides evidence to elucidate the immunotherapeutic role of AhR in cancer, which may be helpful for further functional experiments.

## Materials and Methods

### Data Collection

The genomic and clinicopathological information of 33 cancers were obtained from The Cancer Genome Atlas (TCGA) database (https://portal.gdc.cancer.gov/) and the University of California Santa Cruz Xena Explorer (cohort: TCGA Pan-Cancer). In addition, somatic mutation data were acquired from TCGA database. For the therapeutic cohort, a systematic search was performed to identify the immune checkpoint blockade cohorts, which could be publicly retrieved and reported with complete clinical information. Three immunotherapeutic cohorts were finally employed in this study: advanced urothelial cancer with atezolizumab intervention (IMvigor210 cohort downloaded from previously published research) (14); metastatic melanoma with pembrolizumab treatment (GSE78220 cohort downloaded from the Gene expression omnibus database, GEO), and renal cell carcinoma with nivolumab treatment (GSE67501 cohort downloaded from GEO).

### Clinical Correlation Between AhR Expression and Various Cancers

Using the limma package in R studio software, differential gene expression analysis was performed to determine whether AhR expression varied between tumor and normal groups. The correlation between AhR expression and other clinical parameters (age, gender, and tumor stage) was also investigated. Furthermore, to investigate the time-dependent prognostic value of AhR in 33 cancers, univariate Cox regression analysis was performed using the survival package in R. The considered survival outcomes included overall survival (OS; period from the start of treatment to death from any cause), disease free survival (DFS; period from the start of treatment to disease recurrence or death from any cause), disease specific survival (DSS; cancer survival in the absence of other causes of death), and progression free survival (PFS; period from the start of treatment to disease progression or death from any cause). When the hazard ratio was over 1(HR>1), this indicated that the exposure factor (AhR expression) was the promoting factor of positive events (death). Variations with a *p*-value < 0.05 were considered significant.

### Generation and Investigation of AhR Activity

To further investigate the protein level of AhR in pan-cancers, 69 relevant genes that were significantly up-regulated after 3-MC agonist treatment and down-regulated after GNF-351 antagonist treatment in eight cell lines were identified from a published study (15). AhR activity was generated by single sample gene set enrichment analysis (GSEA). After that, the difference in AhR activity between the normal and tumor groups was investigated. Subsequently, to explore the potential features of AhR expression and activity, the mean value of expression and activity was calculated and arranged in 33 types of cancer.

### Analysis of Potential Association Between AhR Expression and Immune-Related Factors

To begin with, the stromal score and immune score of each case was calculated using the ESTIMATE package; ESTIMATE is a tool for predicting tumor purity and the presence of infiltrating stromal/immune cells in tumor issues (16). The ESTIMATE algorithm is based on single sample GSEA and generates three final scores: the stromal score (indicates the presence of stromal cells in tumor tissues), the immune score (represents the infiltration of immune cells in tumor tissues), and the tumor purity. Subsequently, the abundance of immune cell infiltration in the low AhR-expressing and high AhR-expressing groups was estimated using the CIBERSORT algorithm. CIBERSORT is a deconvolution algorithm that evaluates the proportions of 22 tumor-infiltrating lymphocyte subsets (17). In short, the number of permutations was set to 1000 and the samples in the cohort were eligible for further investigation if they had a p-value < 0.05. Previously published studies indicate that TMB and MSI are closely associated with the immune response. Therefore, the correlation between AhR expression and these indicators was also investigated in this study. TMB was defined as the total number of errors in somatic gene coding, base substitution, gene insertions, or deletions detected in every million bases. To calculate the TMB of each case, the total number of mutations counted was divided by the exome size (38 Mb was utilized as the exome size). The MSI score of each TCGA cancer case was obtained from a previously published study (18). In addition, the underlying relationship between AhR expression and immunological modulators (immune inhibitors, immune stimulators, and MHC molecules) was explored *via* the TISIDB website (http://cis.hku.hk/TISIDB/index.php). The four most relevant results were then highlighted and presented in plots. Finally, to further investigate the relevant signaling pathways, GSEA was performed to identify differential pathways between the low AhR-expressing and high AhR-expressing groups, which were obtained from the Kyoto Encyclopedia of Genes and Genomes database. The relevant signaling pathways were presented in plots if they fulfilled certain criteria (*p* < 0.05) and the pathways with the top five highest normalized enrichment scores were considered.

### Analysis of Immunotherapeutic Response

As previously mentioned, three relevant independent immunotherapeutic cohorts were included and analyzed in this study. In general, immunotherapeutic approaches yielded four outcomes: complete response (CR), partial response (PR), progressive disease (PD), and stable disease (SD). In this research, patients who achieved CR or PR were categorized as responders and compared to non-responders, who showed signs of SD or PD. Then, the Wilcoxon test was utilized to identify differences in AhR expression between the responder and non-responder groups.

## Results

### Clinical Landscape of AhR Expression in 33 Cancers

**Figure 1:**
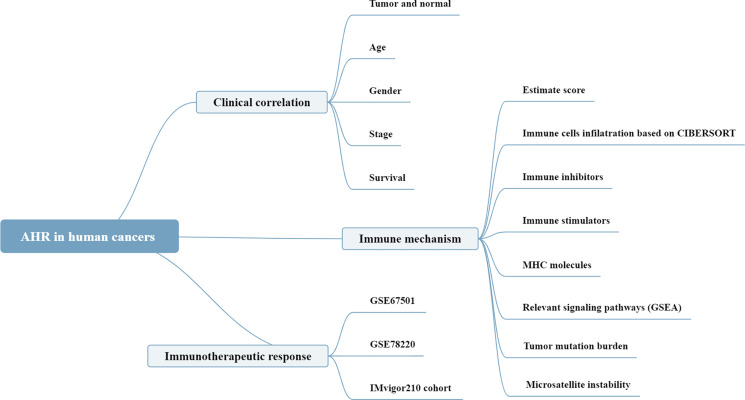
The analyses and indicators employed in our research. In clinical correlation section, differential AhR expression analyses were performed between different tissues (tumor *versus* normal), ages (<=65 *versus* >65), genders (male *versus* female), stages (stage I+II *versus* stage III+IV). Survival correlation analyses were based on univariate Cox regression analysis. In immune mechanism section, relevant signaling pathways were explored by GSEA based on the AhR expression. In immunotherapeutic response section, Wilcoxon test was performed based on the AhR expression of non-responder and responder groups.

**Figure 2:**
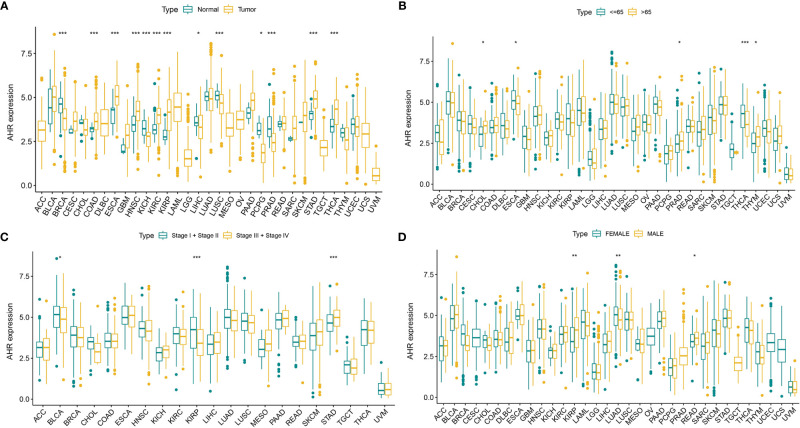
The clinical correlation of AhR. **(A)** shows the differential expression analysis between tumor and normal groups of AhR in 33 cancers; **(B)** represents the correlation between age and AhR; **(C)** demonstrates the correlation between tumor stage and AhR; **(D)** shows the correlation between gender and AhR. “*” indicates *p* < 0.05, “**” indicates *p* < 0.01 and “***” indicates *p* < 0.001.

**Figure 3:**
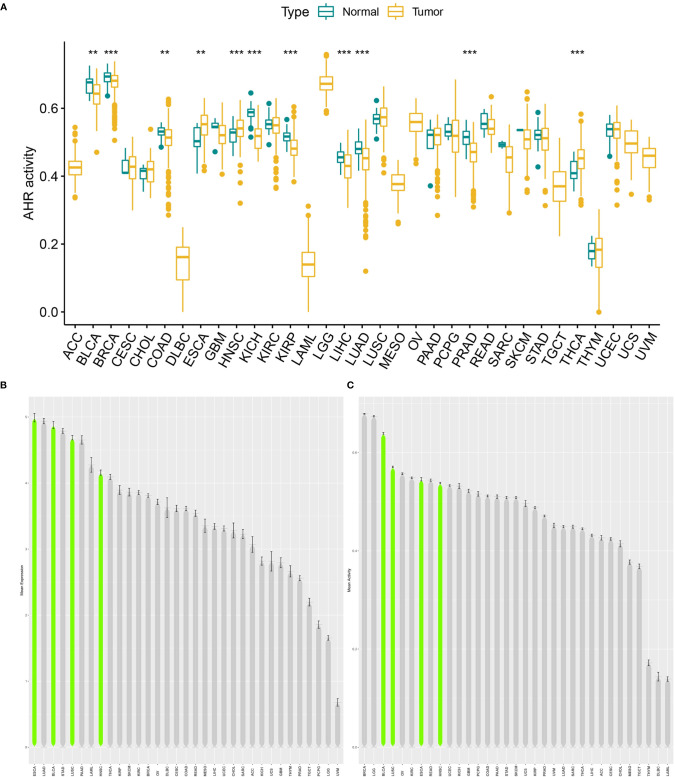
Generation and investigation of AhR activity. **(A)** shows the different activity analysis between tumor and normal groups of AhR in 33 cancers; **(B)** shows the mean expression of AhR in 33 cancers (from high to low); **(C)** shows the mean activity of AhR in 33 cancers (from high to low). “**” indicates p < 0.01 and “***” indicates p < 0.001.

**Figure 4:**
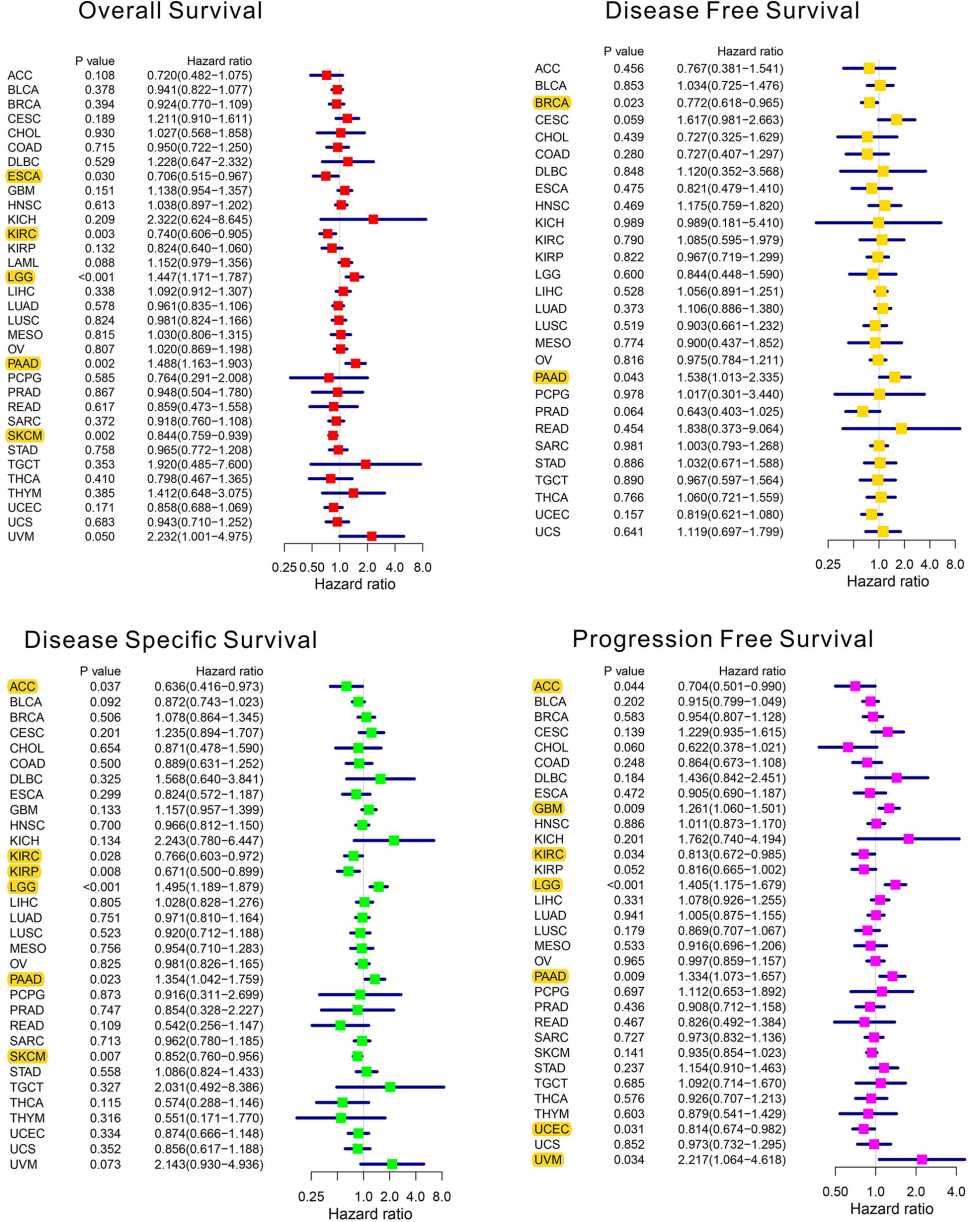
The forest plots of univariate Cox regression analyses. The highlight items mean that AhR expression was significantly correlated with prognosis in these types of cancers (*p* < 0.05). Items with hazard ratio greater than 1 indicated that the AhR expression was a promoting factor of death.

The analysis details are summarized and presented in **Figure 1** for a more comprehensive outlook. The abbreviations and full names of the 33 cancers considered in this study are available in **Table 1**. As illustrated in **Figure 2A**, AhR was differentially expressed in 13 of the 33 cancers (BRCA, COAD, ESCA, HNSC, KICH, KIRC, KIRP, LIHC, LUSC, PCPG, PRAD, STAD, and THCA). AhR was differentially highly expressed among elder patients of the CHOL, PRAD, and THYM groups, whereas it was weakly expressed in ESCA and THCA cases (**Figure 2B**). Meanwhile, AhR expression was significantly correlated with tumor stage of some cancers, including BLCA, KIRP, and STAD (**Figure 2C**). Besides, the results indicated significant gender-based differences in the AhR expression of KIRP, LUAD, and READ (**Figure 2D**). As illustrated in **Figure 3A**, AhR activity was significantly increased in tumor group of ESCA, HNSC and THCA, while decreased in tumor group of BLCA, BRCA, COAD, KICH, KIRP, LIHC, LUAD and PRAD. The results from **Figure 3B, C** demonstrate that four types of cancers (ESCA, BLCA, LUSC and HNSC) exhibit relatively higher expression and activity of AhR. Further, according to the forest plots (**Figure 4**), a positive association was apparent between AhR expression and OS in LGG and PAAD, while a negative association was noted in KIRC, ESCA, and SKCM. Regarding AhR and DFS, a significant negative association was found in BRCA, while a positive association was observed in PAAD. In terms of DSS, AhR expression had a protective effect in ACC, KIRC, KIRP, as well as SKCM, whereas, it seemed to be a risk factor in LGG and PAAD. Moreover, the PFS forest plot confirmed the protective role of AhR expression in ACC, KIRC and UCEC and its role as a risk factor in LGG and PAAD; interestingly, however, using the plot allowed the identification of additional cancers where AhR expression was considered a risk factor, namely GBM and UVM. Although AhR expression was not closely related to clinical parameters, it was strongly associated with survival in some cancers (KIRC, LGG and PAAD).

**Table 1:** 33 types of human cancers employed in our research.

Abbreviation	Full name
ACC	Adrenocortical carcinoma
BLCA	Bladder urothelial carcinoma
BRCA	Breast invasive carcinoma
CESC	Cervical squamous cell carcinoma and endocervical adenocarcinoma
CHOL	Cholangiocarcinoma
COAD	Colon adenocarcinoma
DLBC	Lymphoid neoplasm diffuse large B-cell lymphoma
ESCA	Esophageal carcinoma
GBM	Glioblastoma multiforme
HNSC	Head and neck squamous cell carcinoma
KICH	Kidney chromophobe
KIRC	Kidney renal clear cell carcinoma
KIRP	Kidney renal papillary cell carcinoma
LAML	Acute myeloid leukemia
LGG	Brain lower grade glioma
LIHC	Liver hepatocellular carcinoma
LUAD	Lung adenocarcinoma
LUSC	Lung squamous cell carcinoma
MESO	Mesothelioma
OV	Ovarian serous cystadenocarcinoma
PAAD	Pancreatic adenocarcinoma
PCPG	Pheochromocytoma and paraganglioma
PRAD	Prostate adenocarcinoma
READ	Rectum adenocarcinoma
SARC	Sarcoma
SKCM	Skin cutaneous melanoma
STAD	Stomach adenocarcinoma
TGCT	Testicular germ cell tumors
THCA	Thyroid carcinoma
THYM	Thymoma
UCEC	Uterine corpus endometrial carcinoma
UCS	Uterine carcinosarcoma
UVM	Uveal melanoma

### Underlying Association Between AhR Expression and Immune-Related Factors

**Figure 5:**
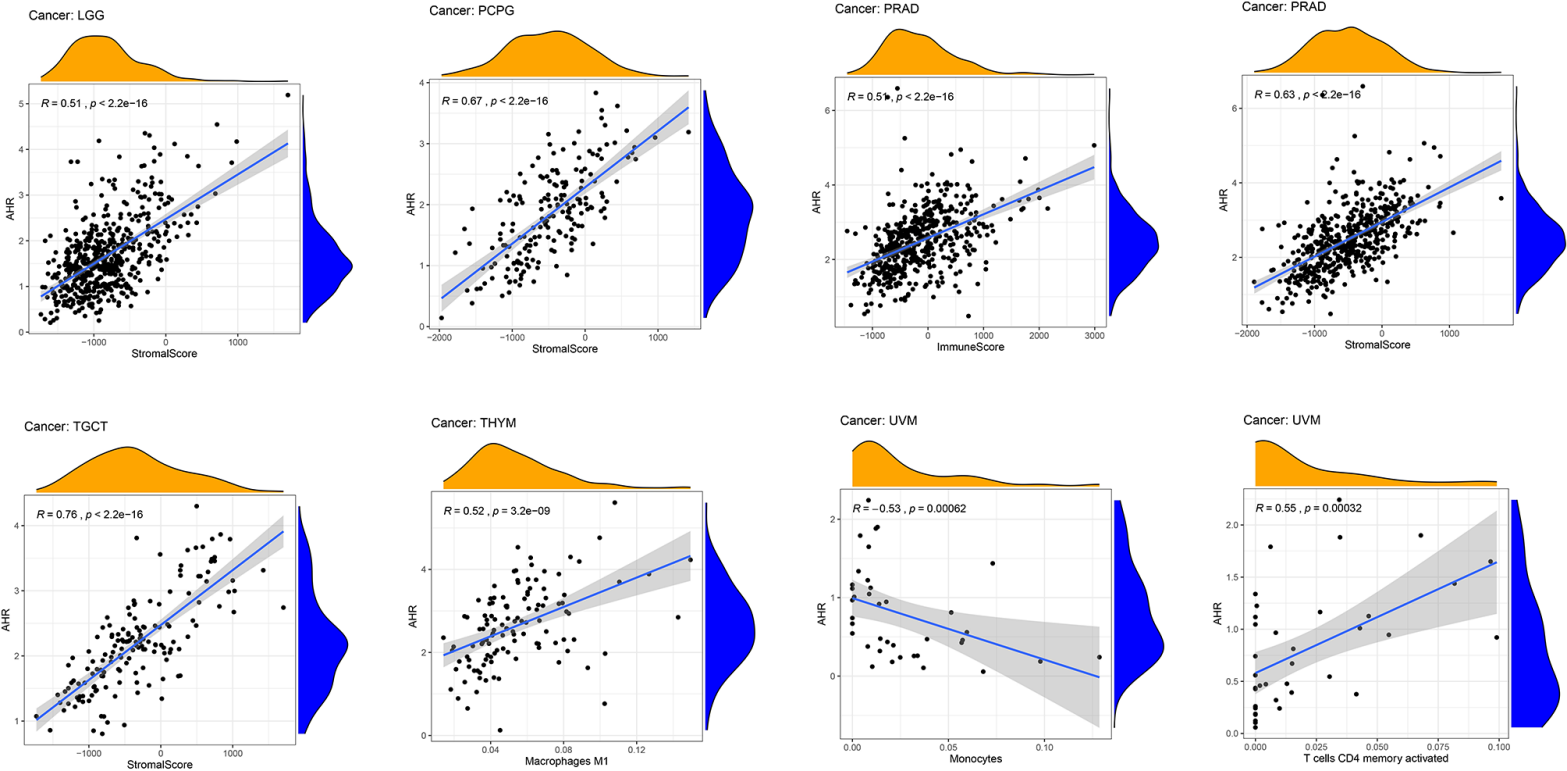
The correlation between AhR expression and both the ESTIMATE score and immune cell infiltration. The ESTIMATE score includes stromal score (indicates the presence of stromal cells in tumor tissues), immune score (represents the infiltration of immune cells in tumor tissues), and tumor purity. The immune cell infiltration was calculated by CIBERSORT algorithm. The correlation plots were illustrated if R > 0.5 and *p* < 0.05.

**Figure 6:**
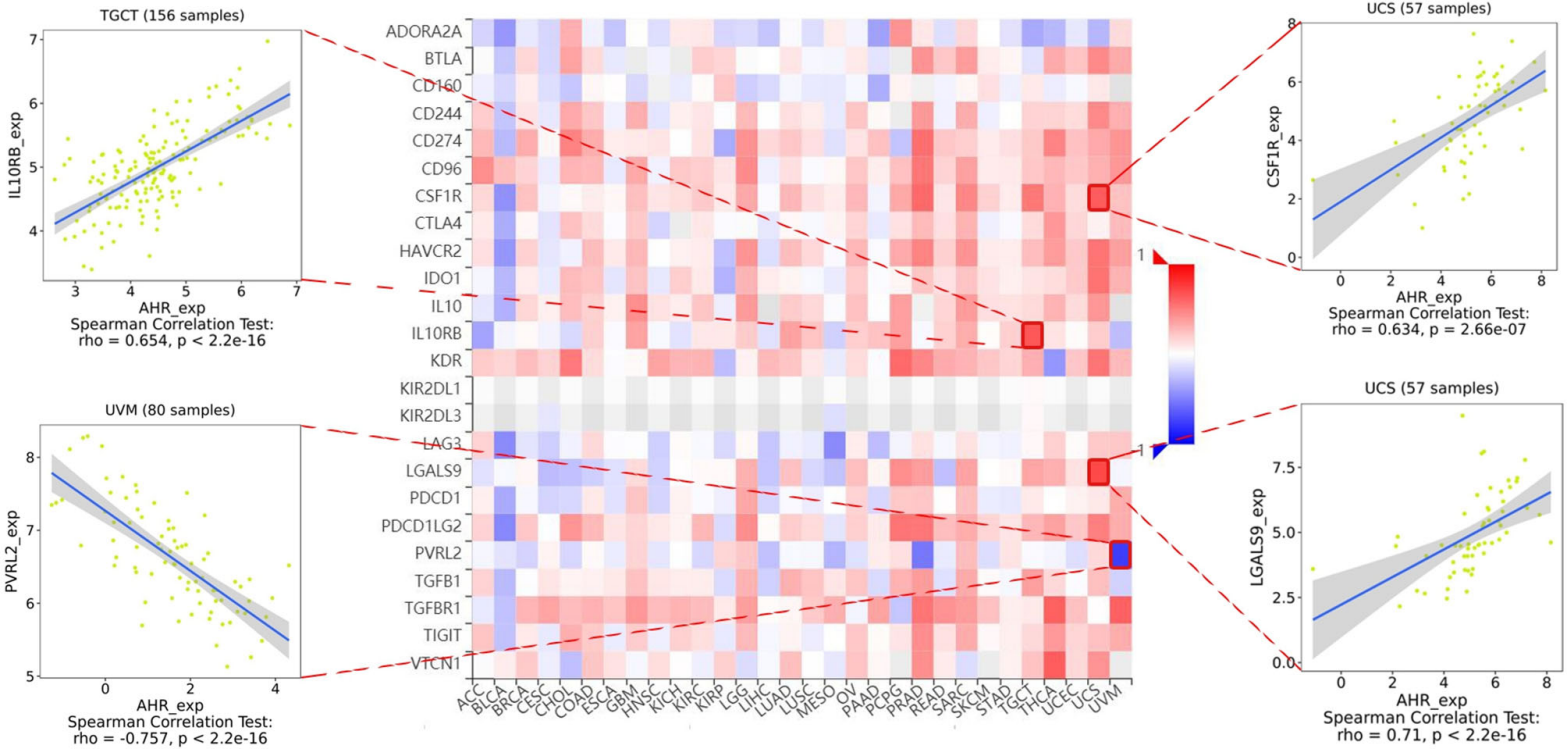
The expression correlation between AhR and immune inhibitors. Red indicates positive correlation whereas blue indicates negative correlation. The top 4 strongest associations were displayed *via* dotplots.

**Figure 7:**
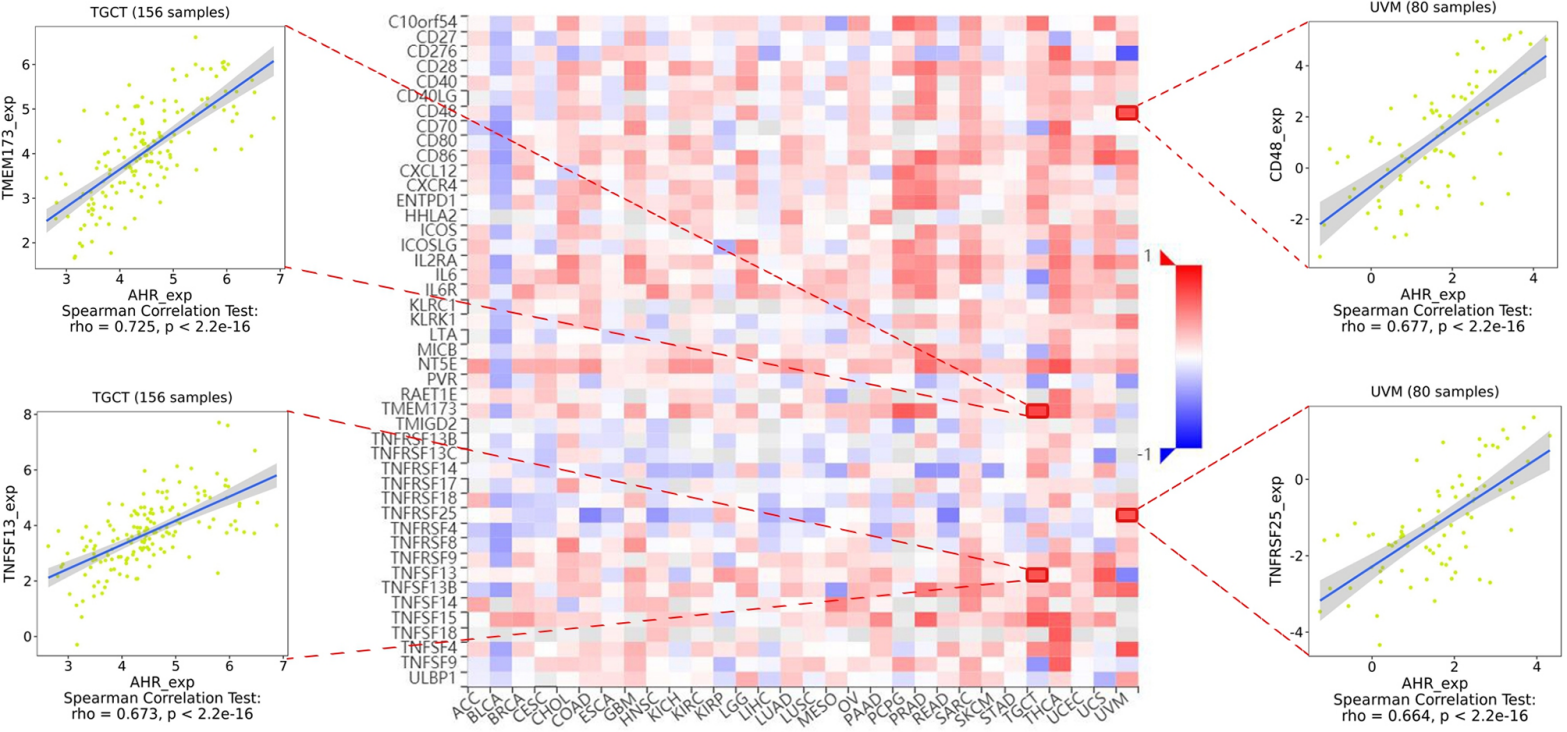
The expression correlation between AhR and immune stimulators. Red indicates positive correlation whereas blue indicates negative correlation. The top 4 strongest associations were displayed *via* dotplots.

**Figure 8:**
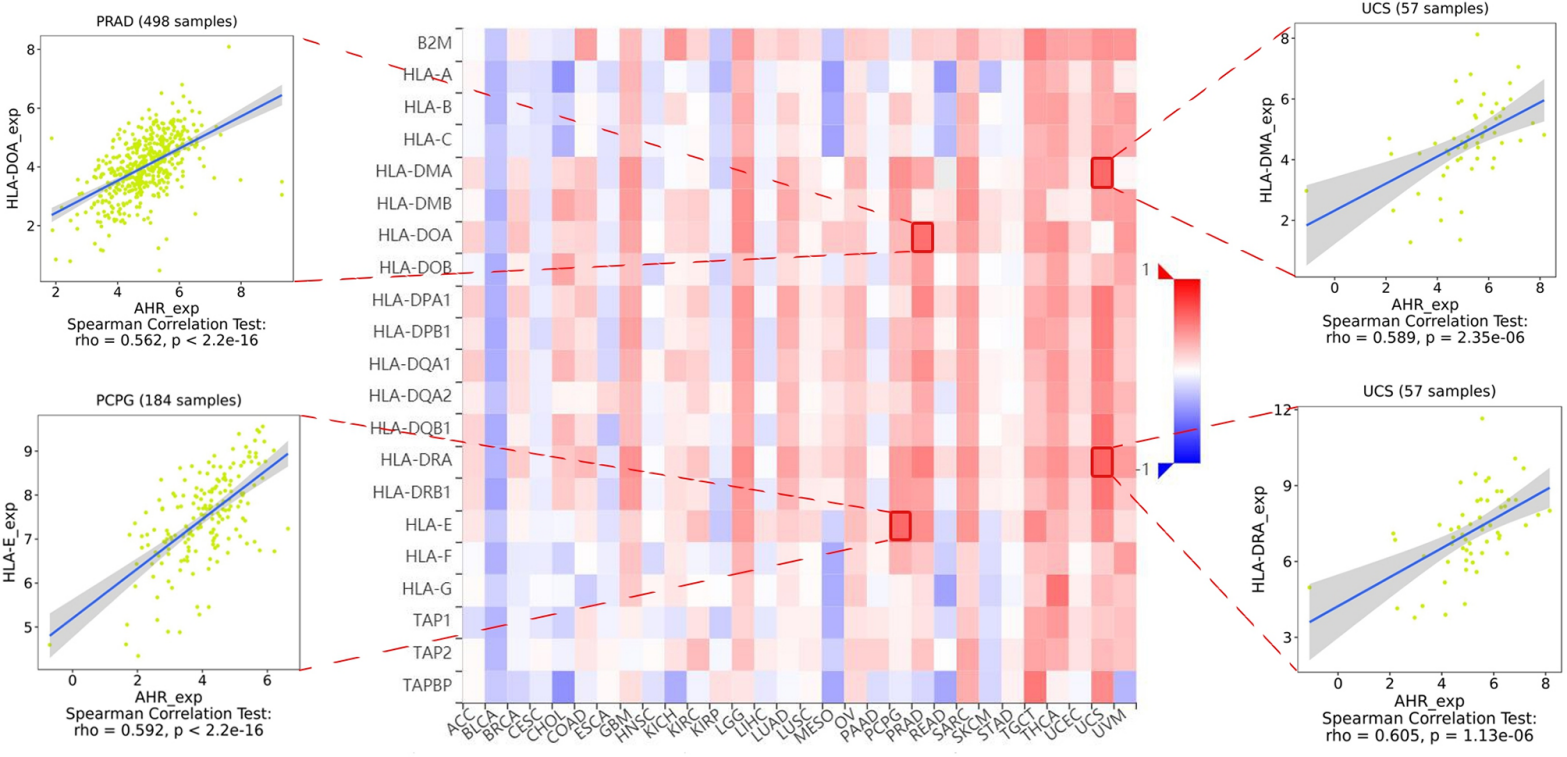
The expression correlation between AhR and MHC molecules. Red indicates positive correlation whereas blue indicates negative correlation. The top 4 strongest associations were displayed *via* dotplots.

The stromal score, immune score, and immune cell infiltration are summarized in **Figure 5** (*p* < 0.01 and |R| > 0.5). Notably, AhR expression was positively associated with the LGG, PCPG, PRAD, and TGCT stromal scores, whereas it was positively related to the PRAD immune score. In terms of immune cell infiltration, AhR expression was positively associated with M1 macrophage content in THYM. In UVM, AhR expression was negatively associated with monocyte infiltration and positively associated with activated CD4^+^ memory T cell infiltration. Furthermore, the correlation between AhR expression and immune modulators was investigated. As illustrated in **Figure 6**, 24 types of immune inhibitors have been analyzed. AhR expression was positively associated with CSF1R and LGALS9 in UCS and IL10RB in TGCT, whereas, it was negatively associated with PVRL2 in UVM. The correlation analyses of 45 immune stimulators (**Figure 7**) demonstrated that AhR expression was positively associated with TMEM173 and TNFSF13 in TGCT as well as CD48 and TNFRSF25 in UVM. Moreover, as shown in **Figure 8**, AhR expression was positively associated with HLA-DOA in PRAD and HLA-E in PCPG. Notably, a positive association was found between AhR expression and both HLA-DMA and HLA-DRA in UCS.

**Figure 9:**
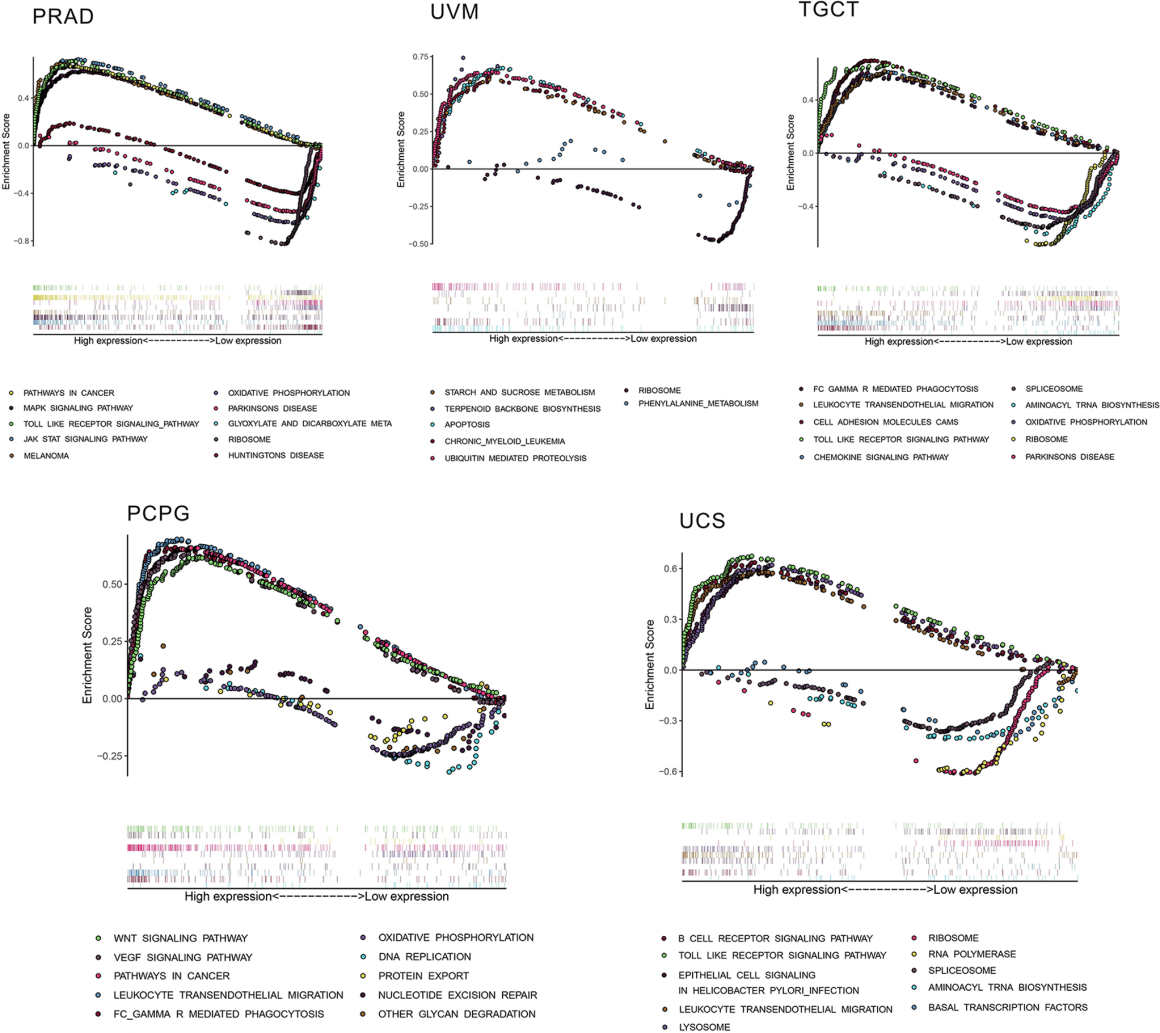
The results of GSEA based on KEGG database. In each panel, the pathways marked in the left were enriched in the high AhR expression group, while the pathways marked in the right were enriched in the low AhR expression group.

**Figure 10:**
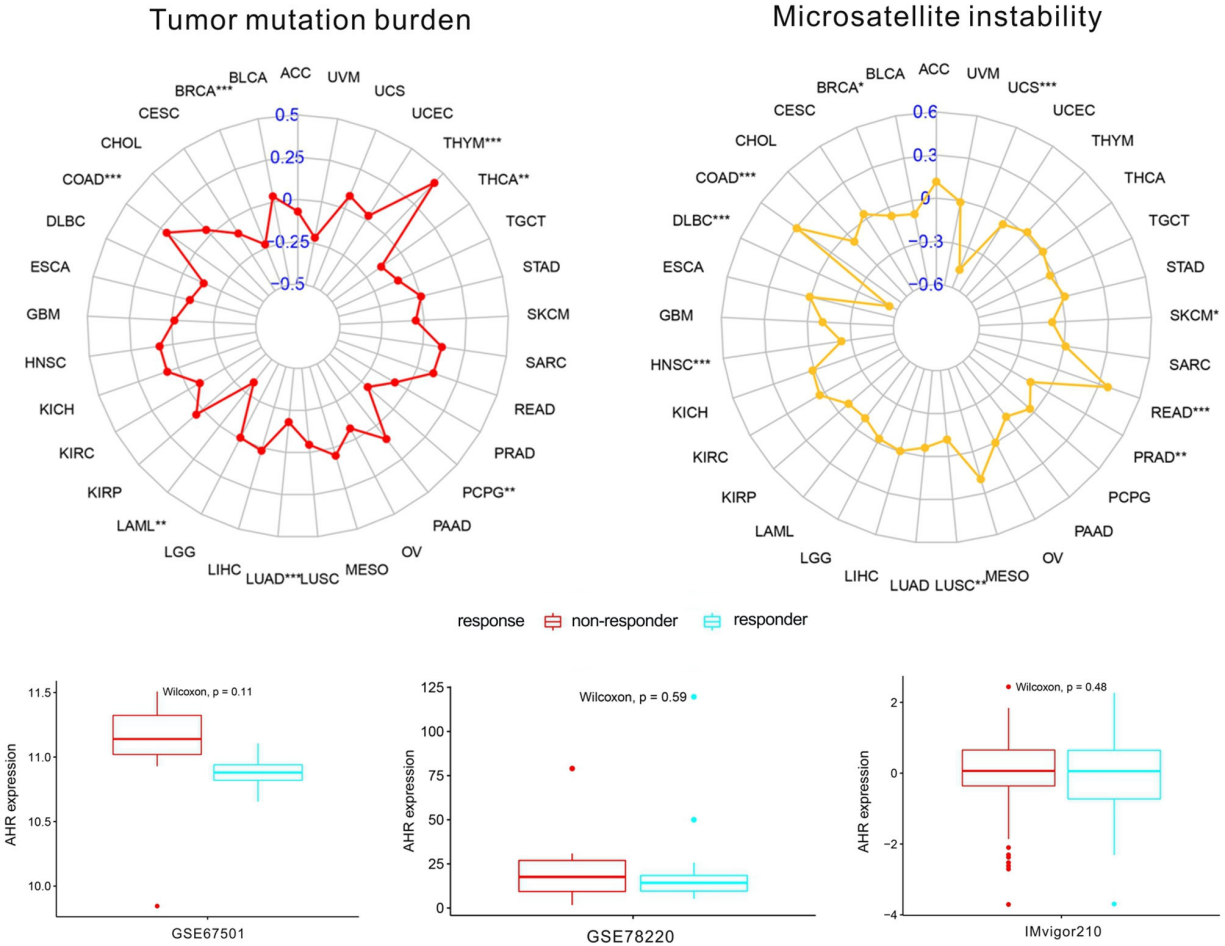
The correlation between AhR and both immunotherapeutic markers and the immunotherapeutic response. “*” indicates *p* < 0.05, “**” indicates *p* < 0.01 and “***” indicates *p* < 0.001.

Considering the robust correlation between AhR and PRAD, UVM, TGCT, PCPG, and UCS, GSEA was performed to investigate the potential pathways involved in AhR signaling in these cancers. The obtained results in **Figure 9** indicate that genes from immune-relevant pathways, such as the Toll like receptor signaling pathway, tend to be enriched in the high-expressing groups of PRAD, TGCT, and UCS. The correlation between AhR and novel dynamic biomarkers of the immune checkpoint blockade (TMB and MSI) were further explored. As presented in **Figure 10**, AhR expression is positively related to the TMB in THYM and COAD, whereas a negative association was observed in THCA, PCPG, LUAD, LAML, and BRCA. For MSI, a positive association in READ and COAD, as well as a negative association in UCS, SKCM, PRAD, LUSC, HNSC, DLBC, and BRCA was identified.

### Immunotherapeutic Response of AhR

As shown in **Figure 10**, no significant difference in AhR expression was evident between the responder and non-responder groups in all three independent cohorts. A trend in the studied cohorts was observed, as patients with low AhR expression were seemingly more responsive to immunotherapy.

## Discussion

The comprehensive investigation of differences in AhR expression between normal and tumor tissues led the discovery of the potential immunotherapeutic value of AhR in various tumors. Contrary to what was initially believed, AhR is not only a toxicant-related transcription factor, but also plays a crucial role in the tumor immune microenvironment. Thus, more AhR-relevant studies involving the tumor microenvironment, immune cells, immune modulators, and the immunotherapeutic response are warranted. In the current study, the aim was to gain more insight regarding the potential mechanisms associating AhR with immune-related factors in 33 human cancers. To begin with, the correlation between AhR and clinical parameters was investigated and no significant differences in age, gender, or tumor stage were identified in most cancer types, which is in accordance with previous findings; Li et al. found that AhR expression is not associated with age, tumor grade, or TNM stage in breast cancer (19). Another study has demonstrated that AhR expression is not related to pathological T stage in renal clear cell carcinoma (20). Interestingly, however, AhR expression had some prognostic value in some cancers, especially PAAD. Similarly, many previous studies have identified AhR as a marker of poor prognosis in various cancers, such as glioblastoma (21), breast cancer (22), and renal clear cell carcinoma (20). In PAAD, the nitric oxide-induced IOD1/Kynurenine/AhR signaling is mediated by RUNX3, a transcription factor, which enhances disease aggressiveness (23). Based on the above evidence, which confirms the usefulness of AhR in cancer prognosis, we hypothesize that therapeutic modulation of AhR activity in various tumor types may be an effective strategy with clinical benefits.

In general, the protein expression level is more able to reflect the tissue activity of AhR. Unfortunately, due to the lack of relevant data in public databases, it is difficult to make relevant analyses at the protein level. However, by comparing the transcriptional level with AhR activity score, in some cancers (BRCA, ESCA, HNSC, KICH, LIHC, PRAD and THCA) the transcription level partially matched the overall AhR activation, indicating that transcription level represented AhR activation in these cancers. Nevertheless, in some cancers (COAD and KIRP), inconsistency was observed between AhR expression and activity. This may be due to post transcriptional protein level modification or protein metabolism affecting AhR expression.

To further investigate the potential value of AhR, the correlation between AhR and immune-cell infiltration was explored. A robust correlation was observed between AhR and M1 macrophages in THYM. Furthermore, previous data suggest that AhR affects tumor development, as well as immune responses within the tumor environment *via* tumor-associated macrophages (12). AhR may play a putative dominant role in macrophage polarization and subsequent induction of an immunosuppressive response (24). In UVM, a negative association was noted in monocytes and a positive association was evident in activated CD4 memory T cells. However, no concrete associations were noted between AhR and immune cells in UVM. Among various immune inhibitors, PVRL2 exhibited the most significant negative association with AhR in UVM. PVRL2 (CD112) is a known ligand of TIGIT, which has recently been identified as a novel checkpoint receptor target in NK cells (25). Current evidence suggests that AhR may affect NK cells *via* regulating inflammatory signaling pathways to induce immune surveillance (12). Based on these data, we propose the existence of an underlying mechanism linking AhR, PVRL2, and NK cells, although this should be verified in functional experiments. For immune stimulators and MHC molecules, most of the modulators exhibited a positive correlation with AhR, with the exception of BLCA; this interesting finding may lead to the discovery of a novel regulatory mechanism in BLCA immunotherapy. Furthermore, Toll-like receptor signaling pathways were enriched in the high AhR-expressing groups of some cancers. Toll-like receptors are transmembrane pattern-recognition receptors that are recognized for their role in innate immunity, particularly in the defense against microbial pathogens (26). The current findings suggested that high AhR expression might modulate innate immunity in some cancers by activating Toll-like receptor pathways.

Additionally, in this study, two immunotherapeutic biomarkers (TMB and MSI) showed a significant association with AhR in some cancers. In general, the more somatic mutations a tumor has, the more neoantigens it is likely to form; the TMB provides a useful estimation of tumor-neoantigen load (27). In contrast, MSI is defined as a robust mutator phenotype caused by deficient DNA mismatch repair and is a potential predictive marker for immunotherapy (28). AhR was negatively correlated with TMB and MSI in BRCA, while it was positively correlated with both biomarkers in COAD. This indicated that AhR might have an indirect effect on the immunotherapeutic response of BRCA and COAD. Subsequently, the correlation between AhR and the immunotherapeutic response was investigated; however, no significant differences were found in either of the studied cohorts. We hypothesize that although the three cohorts underwent and responded to anti-PD1 treatment, AhR may affect the immunotherapeutic response by targeting other immune checkpoints such as CTLA-4 or TIGIT. Meanwhile, only three relevant cohorts were analyzed in our research, which was hard to elucidate the actually immunotherapeutic response of AhR. Studies on more relevant immunotherapeutic cohorts should be performed in the future.

To the best of our knowledge, this is the first study that focuses on the value of AhR in a wide range of cancers (33 types). This research provides valuable insight on the role of AhR in cancer immunotherapy and reveals the association between AhR and important immunological indicators (immune cell infiltration, immune modulators, and immune biomarkers), which may be beneficial for understanding the potential mechanisms linking AhR and the immune system. Although not all the cancers exhibited an association between their tumor immune microenvironment and AhR, these findings highlight the immunological effect of AhR in specific cancers, which will serve as an effective means for targeting them. However, considering that a bioinformatics approach was adopted in this study, these findings are preliminary; thus, more studies on this topic need to be undertaken before the association between AhR and cancer immunotherapy is clearly understood and widely accepted.

## Conclusions

This research is the first to investigate the immunotherapeutic value of AhR in 33 human cancers. We believe that these findings may lay the groundwork for prospective functional experiments and might eventually have an impact in the clinical setting.

## Data Availability Statement

Publicly available datasets were analyzed in this study. This data can be found here: 33 types of cancers from TCGA database (https://portal.gdc.cancer.gov/) GSE78220 and GSE 67501 from GEO database (https://www.ncbi.nlm.nih.gov/geo/) IMvigor210 cohort from published study (doi: 10.1038/nature25501).

## Author Contributions

ZM and SZ designed the manuscript. ZM and PL wrote and completed the manuscript. ZM and ZC completed the data download and analysis. All authors contributed to the article and approved the submitted version.

## Funding

This study was funded by the National Natural Science Foundation of China (NO. 81873248, NO. 81673903).

## Conflict of Interest

The authors declare that the research was conducted in the absence of any commercial or financial relationships that could be construed as a potential conflict of interest.
